# Dynamic Changes in Albumin and Systemic Immune-Inflammation Index as Prognostic Markers in Patients Treated with Cabozantinib After Immune Checkpoint Inhibitors for Metastatic Renal Cell Carcinoma

**DOI:** 10.3390/cancers17243956

**Published:** 2025-12-11

**Authors:** Rikushi Fujimura, Hiroshi Yaegashi, Ryunosuke Nakagawa, Taiki Kamijima, Hiroshi Kano, Tomoyuki Makino, Renato Naito, Suguru Kadomoto, Hiroaki Iwamoto, Kazuyoshi Shigehara, Takahiro Nohara, Kouji Izumi, Atsushi Mizokami

**Affiliations:** Department of Integrative Cancer Therapy and Urology, Graduate School of Medical Science, Kanazawa University, 13-1 Takara-machi, Kanazawa 920-8641, Japan; rikush-f@staff.kanazawa-u.ac.jp (R.F.);

**Keywords:** renal cell carcinoma, cabozantinib, albumin, systemic immune-inflammation index, biomarker, immune checkpoint inhibitor, progression-free survival, dynamic changes

## Abstract

Background/Objectives: Cabozantinib is widely used as subsequent-line therapy after immune checkpoint inhibitor (ICI) treatment in metastatic renal cell carcinoma (mRCC), yet reliable on-treatment biomarkers are lacking. This study explored the prognostic value of a composite score combining early changes in serum albumin (ΔAlb) and the systemic immune–inflammation index (ΔSII) during cabozantinib therapy. Methods: We retrospectively analyzed 40 patients with mRCC who received cabozantinib after prior ICI therapy. Alb and SII were measured at baseline and 6 weeks after initiation. Patients were stratified into three categories according to the ΔAlb + ΔSII composite: both favorable, either unfavorable, or both unfavorable. Progression-free survival (PFS) was analyzed using Kaplan–Meier and Cox regression models. Results: Among 38 evaluable patients, PFS significantly differed across composite categories (*p* for trend < 0.05). Patients with both favorable changes achieved notably longer PFS, while those with both unfavorable changes experienced the shortest. Compared with the both-favorable group, the “either” and “both unfavorable” groups had shorter PFS (HR = 1.83, 95% CI 0.61–5.46; HR = 6.27, 95% CI 1.61–24.49). Conclusions: In this small retrospective cohort, early on-treatment changes in Alb and SII showed an association with PFS in ICI-pretreated mRCC treated with cabozantinib. The ΔAlb + ΔSII composite may serve as a hypothesis-generating framework, warranting confirmation in larger, prospective studies.

## Simple Summary

Systemic inflammation and nutritional status influence outcomes in advanced renal cell carcinoma (RCC). We examined early on-treatment changes in serum albumin (ΔAlb) and the systemic immune–inflammation index (ΔSII) as exploratory dynamic markers during cabozantinib therapy following immune checkpoint inhibitor (ICI) treatment. In this retrospective cohort, a composite of ΔAlb and ΔSII showed an association with progression-free survival, with patients exhibiting favorable changes in both markers tending to experience longer disease control. This simple, laboratory-based composite may provide insight into early host–tumor dynamics during treatment, but its clinical relevance remains exploratory and warrants confirmation in larger, prospective studies.

## 1. Introduction

The therapeutic landscape for metastatic renal cell carcinoma (mRCC) has markedly evolved with the advent of immune checkpoint inhibitor (ICI) combinations and ICI–tyrosine kinase inhibitor (TKI) regimens, which now constitute established first-line standards. Pivotal phase III trials, including CheckMate 214 (nivolumab plus ipilimumab) [1], KEYNOTE-426 (pembrolizumab plus axitinib) [2], JAVELIN Renal 101 (avelumab plus axitinib) [3], and CLEAR (lenvatinib plus pembrolizumab) [4], demonstrated superior overall survival or progression-free survival (PFS) outcomes compared with control-arm therapy, thereby establishing ICI-based combinations as standard first-line options. In addition, the CheckMate 9ER trial confirmed the efficacy of nivolumab plus cabozantinib (Cabo) as a first-line regimen [5], further extending the clinical utility of Cabo across treatment sequences.

Notably, Cabo had originally been validated as a post-TKI therapy in the METEOR trial [6], a phase III randomized controlled study that demonstrated its superiority over everolimus in patients previously treated with one or more TKIs. Given its multitargeted inhibition of vascular endothelial growth factor receptor (VEGFR), MET proto-oncogene, receptor tyrosine kinase (MET), and AXL receptor tyrosine kinase (AXL) pathways, Cabo has become a key agent in both post-TKI and post-ICI settings. However, reliable on-treatment biomarkers that dynamically reflect host–tumor interactions during Cabo therapy remain limited, particularly in the post-ICI era.

In the current treatment paradigm, Cabo remains a widely utilized subsequent-line therapy following progression on ICI-based regimens [7,8,9]. Multiple real-world studies have confirmed its sustained clinical efficacy in the post-ICI setting, with median PFS ranging from approximately 6 to 10 months and objective response rates (ORRs) around 20–30% [10,11,12,13]. Nevertheless, substantial response heterogeneity persists, and the absence of robust predictive biomarkers continues to limit individualized treatment optimization.

Systemic inflammation- and nutrition-related indices such as the neutrophil-to-lymphocyte ratio (NLR), serum albumin (Alb), and systemic immune–inflammation index (SII) have been associated with survival outcomes in mRCC [14,15,16,17,18,19,20,21]. However, most studies have relied solely on baseline measurements, which fail to capture the dynamic biological changes induced by Cabo treatment—such as alterations in tumor–immune interaction, cytokine milieu, and metabolic balance. Given Cabo’s pleiotropic effects on the tumor microenvironment, on-treatment temporal changes (Δ) in host inflammatory and nutritional markers may better reflect treatment response dynamics than static baseline values. Yet, evidence evaluating such early Δ-based biomarkers during Cabo therapy remains scarce.

To address this gap, the present study investigated the prognostic utility of an integrated ΔAlb + ΔSII composite score that incorporates early changes in nutritional (Alb) and inflammatory (SII) status during Cabo therapy in patients with mRCC previously treated with ICI-based regimens.

## 2. Materials and Methods

### 2.1. Study Design and Patients

This retrospective observational study included patients with advanced renal cell carcinoma (RCC) who received Cabo therapy at Kanazawa University Hospital between September 2020 and April 2025. Eligible patients met all of the following criteria: (1) histologically confirmed RCC; (2) received Cabo as second- or later-line therapy; (3) availability of laboratory data on serum Alb and SII both at baseline and at 6 weeks after treatment initiation; and (4) evaluable PFS data.

Patients without post-treatment Alb or SII data were excluded. The study was approved by the institutional review board of Kanazawa University (approval number 114944-1). Because of its retrospective design, the need for individual informed consent was waived. The study was conducted in accordance with the Declaration of Helsinki.

### 2.2. Clinical and Laboratory Data Collection

Demographic and clinicopathological variables included age, sex, histology, International Metastatic RCC Database Consortium (IMDC) risk group, treatment line, and metastatic sites. The IMDC risk group was determined according to the established criteria, classifying patients as favorable, intermediate, or poor risk based on six prognostic factors (Karnofsky performance status < 80%, time from diagnosis to systemic therapy < 1 year, anemia, hypercalcemia, neutrophilia, and thrombocytosis) [22]. Eastern Cooperative Oncology Group (ECOG) performance status was inconsistently documented at Cabo initiation and was therefore excluded from the analyses to avoid selection bias.

Laboratory parameters were collected at baseline and at 6 weeks after Cabo initiation, including Alb, C-reactive protein (CRP), NLR, SII, and lactate dehydrogenase (LDH).

The SII was calculated as platelet count × neutrophil count/lymphocyte count using peripheral blood data. Relative changes were calculated as (value_6_w/value_0_w − 1) × 100 for both Alb (ΔAlb) and SII (ΔSII).

Baseline CRP levels were used in multivariable analyses. The relative dose intensity (RDI all time, %) of Cabo was defined as the ratio of the total delivered to the planned total dose throughout the entire treatment course.

### 2.3. Derivation of ΔAlb and ΔSII Composite Score

For each patient, relative changes in Alb and SII were dichotomized using their respective median values as cut-offs (unfavorable: ΔAlb below the median; ΔSII above the median). A composite score (ΔAlb + ΔSII composite) was then derived as the sum of these indicators: 0 = both favorable, 1 = either unfavorable, and 2 = both unfavorable.

This median-based classification was prespecified and applied consistently across analyses to ensure reproducibility. The derivation process is detailed in the Supplementary Materials.

Relative changes in other inflammation-related markers, including CRP and NLR, were also explored as described in the Supplementary Materials. However, both ΔCRP and ΔNLR showed limited dynamic ranges and weak correlations with treatment outcomes, and were therefore not included in the composite model.

### 2.4. Assessment of Treatment-Related Adverse Events (TRAEs)

Treatment-related adverse events (TRAEs) were graded according to the Common Terminology Criteria for Adverse Events (CTCAE) version 5.0. Events were categorized as early (occurring within 6 weeks after Cabo initiation) or total (occurring at any time during treatment). Grade ≥ 3 events were also identified separately. The detailed distribution of early and total TRAEs is summarized in the Supplementary Materials.

### 2.5. Statistical Analyses

The primary endpoint was PFS, defined as the interval from Cabo initiation to radiographic progression or death from any cause. Univariate Cox proportional hazards models were used to explore associations between each clinical variable and PFS.

Variables with *p* < 0.1 in univariate analyses were entered into a multivariable Cox regression model. In addition, the ΔAlb + ΔSII composite, the IMDC risk group, and RDI during treatment (RDI all time) were included as covariates a priori because of their established clinical relevance as potential confounders of treatment outcomes, even if they did not meet the pre-specified univariate significance threshold.

Kaplan–Meier survival curves were generated according to the composite score, and differences among the three categories (“both favorable,” “either unfavorable,” and “both unfavorable”) were tested using the log-rank trend test. The proportional hazards assumption was examined using scaled Schoenfeld residuals.

Sensitivity analyses were performed to confirm robustness of the findings:

(1) in patients who had received prior ICI therapy only (Prior ICI = 1);

(2) by testing the interaction between composite and Prior ICI;

(3) by excluding the single ICI-naïve patient.

Analyses were performed using R version 4.3.2 (R Foundation for Statistical Computing, Vienna, Austria) and the survival, survminer, and ggplot2 packages, and two-sided *p* < 0.05 was considered statistically significant.

## 3. Results

### 3.1. Patient Characteristics

Baseline patient characteristics are summarized in Table 1. A total of 40 patients with advanced RCC who received Cabo between September 2020 and April 2025 were included in this study. The median age at treatment initiation was 68.0 years (interquartile range [IQR], 59.8–73.3), and 34 patients (85%) were male. Most patients had clear cell histology, and the distribution of IMDC risk groups was as follows: 13 (32.5%) favorable, 22 (55.0%) intermediate, and 5 (12.5%) poor (Figure S1).

Cabo was mainly used as second- or later-line therapy. The median RDI all time was 31.6% (IQR, 22.5–33.5). At baseline, the median Alb was 3.80 g/dL (IQR, 3.20–4.00), CRP was 0.96 mg/dL (IQR, 0.21–5.76), and SII was 1352 (IQR, 678–2170).

ECOG performance status was inconsistently recorded at Cabo initiation and was therefore excluded from the analyses to avoid potential selection bias.

The distribution of the ΔAlb + ΔSII composite score (composite) was as follows: composite = 0 (*n* = 11), composite = 1 (*n* = 21), and composite = 2 (*n* = 6). Two patients were not evaluable for the composite score because Alb was missing at baseline (*n* = 1) or at the 6-week assessment (*n* = 1); therefore, 38 patients were included in the primary composite-based analyses. Detailed laboratory parameter transitions are summarized in Supplementary Table S1.

### 3.2. Changes in Alb and SII After 6 Weeks

At 6 weeks after Cabo initiation, Alb levels decreased in most patients, whereas the SII generally increased compared with baseline values. The relative change (ΔAlb, ΔSII) demonstrated an inverse relationship between nutritional status and systemic inflammation.

**Figure 1:**
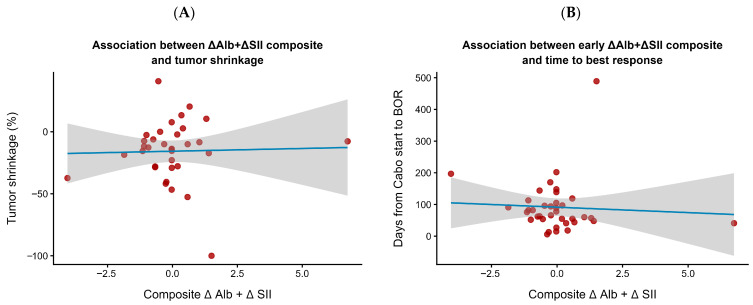
Associations between early ΔAlb + ΔSII composite and treatment response. (**A**) Relationship between the composite index and tumor shrinkage (%). (**B**) Relationship between the composite index and the time to best overall response (BOR). The composite index (ΔAlb + ΔSII) was calculated to integrate early systemic inflammatory and nutritional changes at 6 weeks after cabozantinib initiation. A weak trend suggested that patients with early improvement in inflammation and nutritional status (lower composite values) tended to show greater and earlier tumor responses. Each dot represents an individual patient. The solid blue line indicates the fitted linear regression, and the shaded area represents the 95% confidence interval.

As illustrated in Figure 1A, larger relative declines in Alb tended to be observed in patients with less tumor shrinkage, although the correlation was modest, indicating an exploratory association rather than a definitive relationship. Similarly, Figure 1B shows that patients with both unfavorable changes (ΔAlb below and ΔSII above the median) tended to reach their best overall response earlier, a pattern consistent with more rapid disease progression rather than durable treatment control.

Overall, these observations highlight exploratory trends in early Alb and SII dynamics, rather than establishing predictive or causal relationships with treatment efficacy.

### 3.3. PFS According to the ΔAlb + ΔSII Composite Score

**Figure 2:**
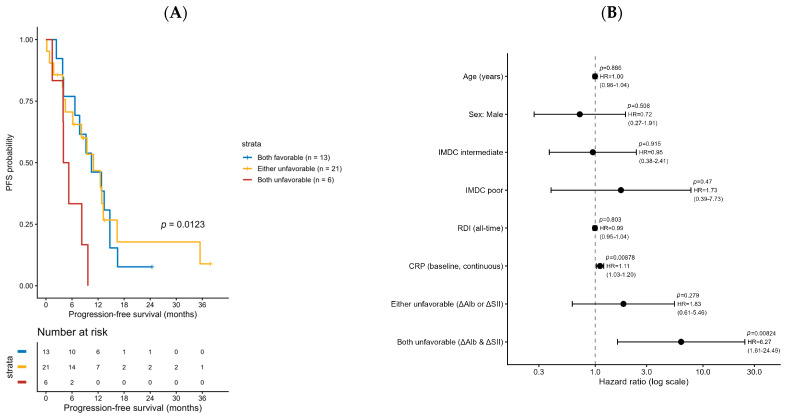
Kaplan–Meier curves for progression-free survival (PFS) stratified by the composite ΔAlb + ΔSII score. (**A**) Patients were classified into three groups: both favorable (blue), either unfavorable (orange), and both unfavorable (red). A trend toward shorter PFS was observed with an increasing number of unfavorable changes (log-rank test for trend, *p* = 0.0123). (**B**) Multivariate Cox proportional hazards analysis for progression-free survival (PFS). The model included age, sex, IMDC risk category, relative dose intensity (RDI), baseline C-reactive protein (CRP), and the combination group based on ΔAlb and ΔSII changes. The “both unfavorable” group (ΔAlb & ΔSII) was independently associated with shorter PFS (HR = 6.27, 95% CI 1.61–24.49, *p* = 0.008), while the “either unfavorable” group (ΔAlb or ΔSII) showed a non-significant trend (HR = 1.83, 95% CI 0.61–5.46, *p* = 0.279). Baseline CRP level was also significantly associated with shorter PFS (HR = 1.11 per unit increase, 95% CI 1.03–1.20, *p* = 0.009).

PFS differed markedly according to the ΔAlb + ΔSII composite score. As shown in Figure 2A, patients with both favorable changes (composite = 0) achieved the longest median PFS, whereas those with both unfavorable changes (composite = 2) experienced the shortest. The PFS trend across the three categories (“both favorable,” “either unfavorable,” and “both unfavorable”) was statistically significant (log-rank test for trend, *p* = 0.0123).

In the multivariable Cox proportional hazards model (Figure 2A, Table 2 and Table S2), the composite score remained an independent prognostic factor for PFS after adjustment for age, sex, IMDC risk group, RDI all time, and baseline CRP. 

Compared with the reference group (both favorable), the adjusted hazard ratios were 1.83 (95% CI, 0.61–5.46; *p* = 0.279) for the “either unfavorable” group and 6.27 (95% CI, 1.61–24.49; *p* = 0.008) for the “both unfavorable” group.

Exploratory analyses of other inflammation-related markers, including CRP and NLR, are shown in the Supplementary Materials (Supplementary Figure S2). Both ΔCRP and ΔNLR exhibited minimal variability and were therefore not incorporated into the composite model. Because NLR is mathematically included within the SII formula, it was also excluded from the multivariable Cox proportional hazards analysis to avoid collinearity.

Other covariates, including Age, Sex, IMDC risk group, and RDI, were not significantly associated with PFS. The proportional hazards assumption was not violated (global PH test, *p* = 0.587; Supplementary Figure S3).

In post hoc pairwise log-rank comparisons, PFS was significantly shorter in patients with a composite score of 2 than in those with a score of 0, with this difference remaining significant after Holm adjustment, whereas differences involving the intermediate group (composite score 1) were attenuated after multiple-comparison.

### 3.4. Sensitivity and Exploratory Analyses

Sensitivity analyses were performed to assess the robustness of the findings.

First, when restricted to patients who had received prior ICI therapy (*n* = 39), the ΔAlb + ΔSII composite score remained significantly associated with progression-free survival (Supplementary Table S3).

Second, the interaction term between the composite score and prior ICI exposure (composite × Prior ICI) was not statistically significant, indicating that the prognostic effect of the composite was independent of prior ICI therapy.

Third, exclusion of the single ICI-naïve case did not materially alter the results.

Additionally, an exploratory analysis was conducted to evaluate the internal consistency of Alb change metrics (Supplementary Figure S3). The relative (ΔAlb %) and absolute (ΔAlb g/dL) changes were highly correlated. Among the 38 evaluable patients, 9 met both the relative (≤−4%) and absolute (≤−0.2 g/dL) decrease thresholds, 2 met either, and 29 met neither.

These results confirm the internal validity and robustness of ΔAlb-based classification within the composite index.

### 3.5. Treatment-Related Adverse Events

Treatment-related adverse events (TRAEs) were common during Cabo therapy and are summarized in Supplementary Figure S4A,B. Early TRAEs (≤6 weeks after treatment initiation) were observed in 32 of 40 patients (80%), and any-grade events during the total treatment course occurred in 36 patients (90%).

The most frequent early TRAEs were decreased appetite (42.5%), fatigue (35.0%), and diarrhea (30.0%), which largely overlapped with events occurring over the entire treatment period. Grade ≥ 3 events occurred in 6 patients (15%) within the first 6 weeks and in 10 patients (25%) overall. The most common severe events were fatigue, hypertension, and palmar–plantar erythrodysesthesia.

No treatment-related deaths occurred. Most TRAEs were manageable with dose reductions or temporary treatment interruptions, and no unexpected safety signals were identified compared with previous clinical experience with Cabo (Supplementary Figure S5).

### 3.6. Internal Validation by Bootstrap Resampling

To evaluate the internal validity and potential overfitting of the multivariable Cox model, bootstrap resampling was performed with 1000 iterations. The calibration curve comparing the predicted and observed 12-month PFS probabilities demonstrated good agreement (Supplementary Figure S6).

The bootstrap-corrected calibration line closely approximated the ideal diagonal, indicating minimal optimism in model performance. The apparent and bias-corrected estimates showed only minor deviation, suggesting that the model retained stable predictive accuracy after internal validation.

These results support the robustness and generalizability of the multivariable Cox model for predicting PFS based on early changes in Alb and SII during Cabo therapy.

## 4. Discussion

In this study, we evaluated early dynamic changes in Alb (ΔAlb) and SII (ΔSII) during Cabo therapy in patients with mRCC previously treated with ICIs. Using an exploratory, hypothesis-generating approach, univariate and multivariable Cox regression analyses demonstrated that the ΔAlb + ΔSII composite score was associated with PFS, remaining significant after adjustment for age, sex, IMDC risk group, RDI, and baseline CRP (HR for both unfavorable vs. both favorable = 6.27, 95% CI 1.61–24.49). Although survival differences were most pronounced between the extreme categories, a consistent risk gradient was observed across the three groups (*p* for trend = 0.012).

Previous studies have reported the prognostic value of baseline inflammatory and nutritional markers such as NLR, SII, and Alb in mRCC treated with TKIs or ICIs [11,12,13,14]. However, few have explored early on-treatment dynamics or integrated composite changes in these parameters. To the best of our knowledge, this is among the first studies to incorporate ΔAlb and ΔSII into a unified prognostic framework in the post-ICI Cabo setting.

Biologically, the SII integrates neutrophil and platelet counts relative to lymphocytes, reflecting the balance between pro-tumor inflammation and anti-tumor immunity—components that overlap with IMDC prognostic factors (neutrophilia, thrombo-cytosis). Cabo, a multitarget TKI, has been shown to reduce myeloid-derived suppressor cells (MDSCs), normalize tumor vasculature, and enhance effector T-cell trafficking [23,24,25,26]. A decline in SII during treatment may therefore reflect immunomodulatory effects, though this remains a hypothesis requiring direct validation. Similarly, early declines in Alb may capture tumor-driven catabolic stress or impaired host resilience [27], but causality cannot be inferred from this observational study.

The ΔAlb + ΔSII composite relies solely on routine laboratory parameters—Alb and complete blood counts—enabling early on-treatment assessment at 6 weeks. Treatment-related adverse events were comparable across composite categories (Supplementary Figure S3), suggesting that early deteriorations in Alb or SII likely reflect disease biology rather than treatment toxicity.

Exploratory internal validation using bootstrap resampling (B = 1000) yielded an optimism-corrected concordance index (C-index) of 0.61, indicating limited but above-random predictive discrimination despite the small sample size (*n* = 38) (Figure 2A,B). This preliminary performance does not support immediate clinical application but justifies further investigation in larger, prospective cohorts to assess reproducibility and clinical utility.

From a translational perspective, concordant unfavorable changes in Alb and SII may represent tumor-driven systemic stress and immune suppression, whereas favorable dynamics could suggest preserved host integrity. Exploratory analyses also showed high correlation between relative and absolute Alb changes (Supplementary Figures S5 and S6), supporting the robustness of ΔAlb as a continuous marker.

Systemic inflammatory indices like SII and NLR have been reported to dynamically reflect immune restoration during effective therapy in mRCC and other cancers [28,29]. Hypoalbuminemia, beyond nutritional depletion, reflects tumor-induced acute-phase responses mediated by proinflammatory cytokines [30,31]. Integrating ΔAlb and ΔSII thus provides a composite view of host–tumor metabolic and immunologic interplay, offering a mechanistically plausible—though not yet validated—framework for monitoring treatment adaptation.

In summary, this small retrospective single-center analysis suggests that early combined changes in Alb and SII are associated with PFS during Cabo therapy following ICIs. The ΔAlb + ΔSII composite is derived from routinely available laboratory tests and demonstrates preliminary prognostic discrimination; however, the findings should be interpreted strictly as exploratory. Prospective validation in larger, independent cohorts is essential before considering any role in risk stratification or treatment decision-making.

## 5. Conclusions

Early on-treatment changes in Alb and SII (ΔAlb + ΔSII composite) were associated with PFS in ICI-pretreated mRCC patients receiving Cabo, with a significant risk gradient across composite categories (*p* for trend = 0.012). Exploratory internal validation yielded an optimism-corrected C-index of 0.61, indicating limited but above-random discrimination. This simple, laboratory-based composite shows preliminary promise as a dynamic prognostic tool but requires prospective validation in larger cohorts before clinical consideration.

## Figures and Tables

**Table 1:** Baseline characteristics of patients (*n* = 40).

Variable	*n* (%)
Age at cabozantinib initiation, years	68.00 [59.75–73.25] *
Sex	
Male	34(85.0)
Female	6 (15.0)
Histology	
Clear cell	36 (90.0)
Non-clear cell	4 (10.0)
IMDC risk group	
Favorable	13 (32.5)
Intermediate	22 (55.0)
Poor	5 (12.5)
Treatment line of cabozantinib	
Second line	12 (30.0)
≥Third line	28 (70.0)
Prior nephrectomy	33 (82.5)
Prior ICI exposure	39 (97.5)
Prior TKI exposure	36 (87.5)
** Metastatic sites	
Lung	31 (77.5)
Lymph node	19 (47.5)
Liver	16 (40.0)
Bone	11 (27.5)
Pancreas	3 (7.5)
Pleura	2 (5.0)
Brain	1 (2.5)
Baseline serum albumin (g/dL)	3.80 [3.20–4.00] *
Baseline CRP (mg/dL)	0.96 [0.21–5.76] *
Baseline NLR	4.60 [3.25–6.82] *
Baseline SII	1352 [678–2170] *
*** Baseline LDH (U/L)	189 [135–252] *
RDI all time	31.59 [22.49–33.47] *
Starting dose of cabozantinib (mg/day)	20 [20–40] *
Dose reduction within 6 weeks	20 (50.0)
Progression-free survival (months)	8.4 [4.0–12.9] *

CRP, C-reactive protein; ICI, immune checkpoint inhibitor; IMDC, International Metastatic RCC Database Consortium; LDH, Lactate dehydrogenase; NLR, Neutrophil-to-lymphocyte ratio; RDI, Relative dose intensity; SII, Systemic immune–inflammation index; TKI, tyrosine kinase inhibitor. * Values are presented as median [IQR]. ** Patients may have multiple metastatic sites. *** LDH values were converted to IFCC units using the formula *IFCC* = 0.535 × *JSCC* + 9.

**Table 2:** Multivariate Cox proportional hazards model for progression-free survival.

Variable	Category/Description	HR (95% CI)	*p*-Value	PH Assumption (*p*)
Age	Continuous	1.00 (0.96–1.04)	0.886	0.707
Sex	Male vs. Female	0.72 (0.27–1.91)	0.508	
IMDC risk group	Intermediate vs. Favorable	0.95 (0.38–2.41)	0.915	
	Poor vs. Favorable	1.73 (0.39–7.73)	0.470	
RDI all time (%)	Continuous	0.99 (0.95–1.04)	0.803	0.529
CRP (mg/dl)	Continuous	1.11 (1.03–1.20)	0.00878	0.65
ΔAlb + ΔSII composite		Reference	-	-
	1 (Either unfavorable)	1.83 (0.61–5.46)	0.279	
	2 (Both unfavorable)	6.27 (1.61–24.49)	0.00824	
Global PH assumption test				0.587

CI, Confidence interval; CRP, C-reactive protein; ΔAlb, change in serum albumin; ΔSII, change in systemic immune–inflammation index; HR, hazard ratio; IMDC, International Metastatic RCC Database Consortium; PH, proportional hazards; RDI, relative dose intensity (Global PH test *p* = 0.587).

## Data Availability

The data presented in this study are available on request from the corresponding author. The data are not publicly available due to privacy restrictions.

## Supplementary Materials

The following supporting information can be downloaded at: https://www.mdpi.com/article/10.3390/cancers17243956/s1, Figure S1: Derivation of ΔAlb + ΔSII composite score; Figure S2: Distribution of ΔCRP_rel and ΔNLR_rel in the study cohort; Figure S3: Proportional hazards (PH) assumption test for the ΔAlb + ΔSII composite model; Figure S4: Treatment-related adverse events; Figure S5: Relationship between relative and absolute changes in serum albumin (ΔAlb); Figure S6: Internal validation of the multivariable Cox proportional hazards model by bootstrap resampling (B = 1000); Table S1: Laboratory parameter changes from baseline to 6 weeks; Table S2: Univariate Cox proportional hazard model; Table S3: Sensitivity analysis of PFS according to ΔAlb + ΔSII composite (Prior ICI only vs. all patients); Table S4: Concordance between relative and absolute albumin decrease criteria.

## Abbreviations

The following abbreviations are used in this manuscript:

Alb	Serum albumin
AXL	AXL receptor tyrosine kinase
BOR	Best overall response
Cabo	Cabozantinib
CI	Confidence interval
CRP	C-reactive protein
Δ	Change from baseline
HR	Hazard ratio
ICI	Immune checkpoint inhibitor
IMDC	International Metastatic RCC Database Consortium
KM	Kaplan–Meier
LDH	Lactate dehydrogenase
mRCC	Metastatic renal cell carcinoma
NLR	Neutrophil-to-lymphocyte ratio
PFS	Progression-free survival
RDI	Relative dose intensity
SII	Systemic immune–inflammation index
TKI	Tyrosine kinase inhibitor
TRAE	Treatment-related adverse event
