# Supplementary material for: Dynamic Changes in Albumin and Systemic Immune-Inflammation Index as Prognostic Markers in Patients Treated with Cabozantinib After Immune Checkpoint Inhibitors for Metastatic Renal Cell Carcinoma
Source: Cancers (Basel). 2025 Dec 11;17(24):3956. doi: 10.3390/cancers17243956 (PMC12730239; doi:10.3390/cancers17243956)
Supplement: Supplementary file 1 [file cancers-17-03956-s001.zip › cancers-4003871-supplementary.pdf]

cancers

MDPI

Supplementary Materials

Table S1. Laboratory parameter changes from baseline to 6 weeks.

Parameter	Baseline	Week6	AbsDelta	RelDelta
Albumin (g/dL)	3.80 [3.20–4.00]	3.60 [3.10–4.00]	0.00 [-0.17–0.27]	0.0 [-4.8–7.1]
CRP (mg/dL)	0.96 [0.21–5.76]	0.77 [0.28–3.81]	0.04 [-0.85–0.61]	4.4 [-61.7–122.9]
NLR	4.60 [3.25–6.82]	2.29 [1.51–3.68]	-1.84 [-3.43–-0.70]	-40.8 [-61.4–-23.0]
SII (×10^4)	1351.93 [677.83–2169.97]	492.31 [294.23–980.78]	-615.22 [-1356.40–-239.90]	-54.1 [-72.5–-31.8]

AbsDelta, Absolute change from baseline; CRP, C-reactive protein; NLR, Neutrophil-to-lymphocyte ratio; RelDelta, Relative change in baseline; SII, Systemic immune–inflammation index.

Table S2. Univariable Cox proportional hazards analysis for progression-free survival.

Variable	Category / Description	HR (95% CI)	*p*-value
Age at cabozantinib initiation (years)	Continuous	1.00 (0.97–1.04)	0.784
Sex	Male vs. Female	0.58 (0.24–1.42)	0.232
IMDC risk group	Intermediate vs. favorable	1.06 (0.47–2.37)	0.896
	Poor vs. favorable	2.59 (0.85–7.87)	0.094
Relative dose intensity (all time)	Continuous	0.99 (0.97–1.02)	0.716
Baseline CRP (mg/dL)	Continuous	1.07 (1.01–1.14)	0.020
Δ Alb or ΔSII composite	0 (Both favorable)	Reference	-
	1 (Either unfavorable)	0.95 (0.44–2.06)	0.894
	2 (Both unfavorable)	3.09 (1.08–8.88)	0.036

CI, Confidence interval; CRP, C-reactive protein; ΔAlb, change in serum albumin; ΔSII, change in systemic immune inflammation index; HR, hazard ratio; IMDC, International Metastatic RCC Database Consortium.

Table S3. Sensitivity analysis of progression-free survival according to ΔAlb + ΔSII composite (Prior ICI only vs. all patients).

Model	Variable	HR	Lower 95% CI	Upper 95% CI	*p*-value
All patients	Δ Alb + ΔSII composite	1.8366148	0.9889950	3.410688	0.05423876
All patients	IMDC (intermediate)	1.2221137	0.5315472	2.809839	0.63677931
All patients	IMDC (poor)	3.2715051	0.8578324	12.476499	0.08266377
All patients	RDI all time	0.9938241	0.9577143	1.031295	0.74286071
Prior ICI only	Δ Alb + ΔSII composite	1.8434837	0.9639672	3.525464	0.06445395
Prior ICI only	IMDC (intermediate)	1.1930770	0.5075276	2.804641	0.68562037
Prior ICI only	IMDC (poor)	3.1325613	0.8071161	12.158028	0.09888897
Prior ICI only	RDI all time	0.9944062	0.9584783	1.031681	0.76511262

CI, confidence interval; ΔAlb, change in serum albumin; ΔSII, change in systemic immune–inflammation index; HR, hazard ratio; ICI, immune checkpoint inhibitor; IMDC, International Metastatic RCC Database Consortium; RDI, relative dose intensity.

Table S4. Concordance between relative and absolute albumin decrease criteria.

Group	n	Percent
Both	9	22.5

Group	n	Percent
Either	2	5.0
Neither	29	72.5

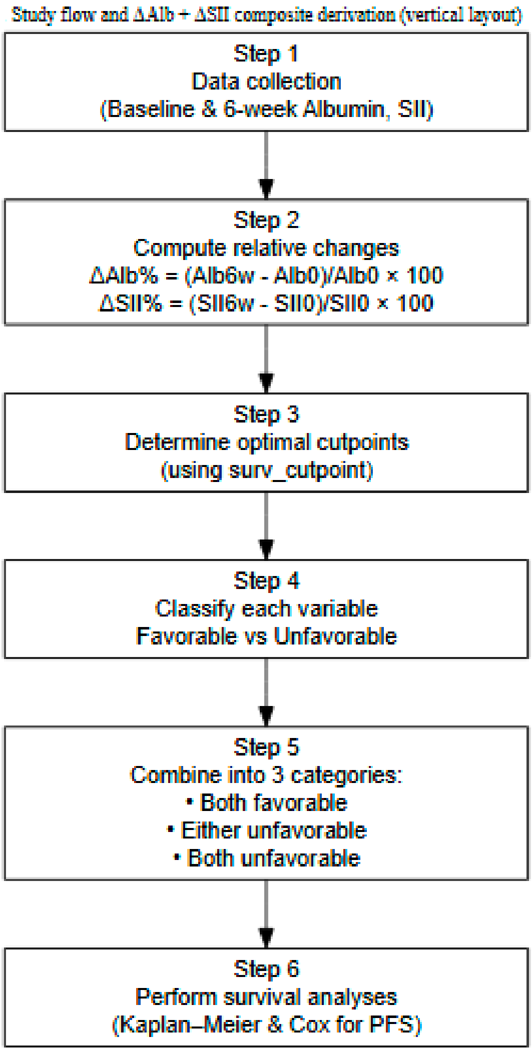

Figure S1. Derivation of ΔAlb + ΔSII composite score. Schematic diagram showing the analytic process of the study. Changes in serum albumin (ΔAlb) and systemic immune-inflammation index (ΔSII) were calculated between baseline and post-treatment assessments. Optimal cutpoints for ΔAlb and ΔSII were determined by maximally selected rank statistics. Patients were classified into three groups based on the combination of ΔAlb and ΔSII status (both favorable, either unfavorable, both unfavorable). The composite variable was then used for Kaplan–Meier and Cox analyses.

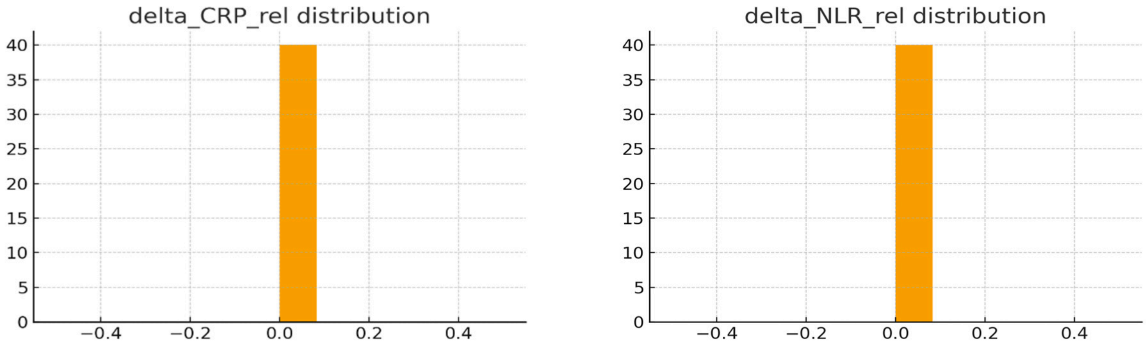

Figure S2. Distribution of ΔCRP_rel and ΔNLR_rel in the study cohort. Histograms showing relative change distributions of CRP (ΔCRP_rel) and NLR (ΔNLR_rel) between baseline to 6 weeks of cabozantinib therapy. Both ΔCRP and ΔNLR exhibited minimal variability and were therefore not incorporated into the composite model. Because NLR is mathematically included within the SII formula, it was also excluded from the multivariable Cox proportional hazards analysis to avoid collinearity.

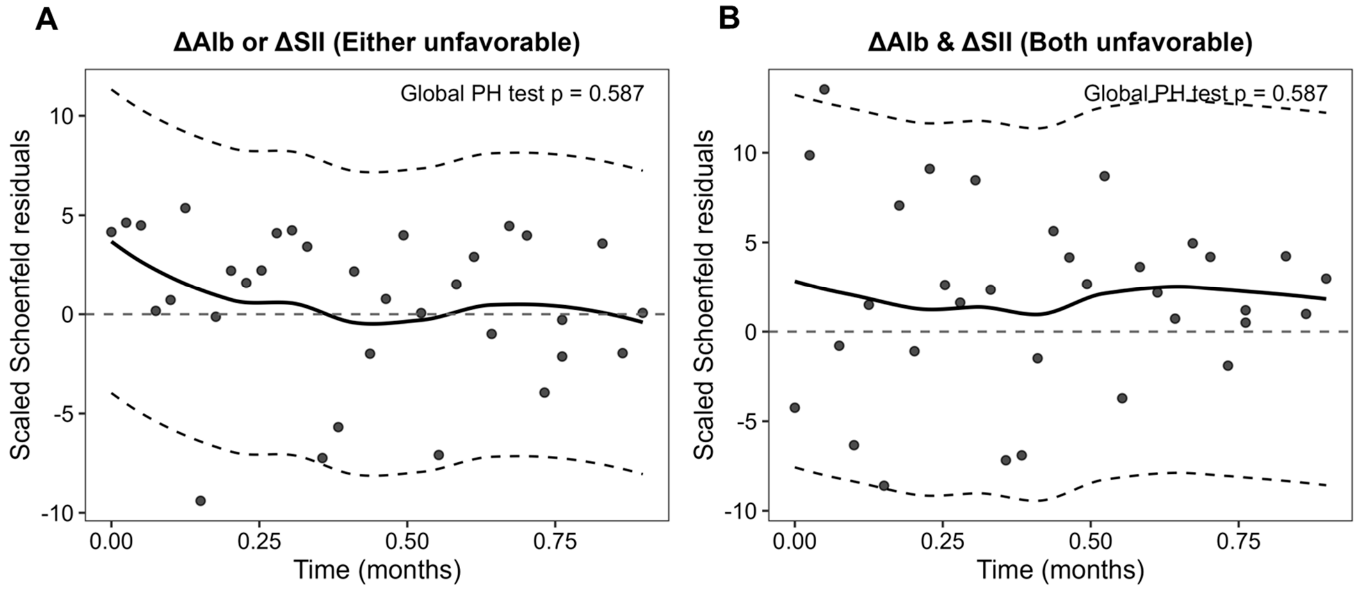

Figure S3. Proportional hazards (PH) assumption test for the ΔAlb + ΔSII composite model **(A)** ΔAlb or ΔSII (Either unfavorable); **(B)** ΔAlb and ΔSII (Both unfavorable). The solid line represents the fitted smooth curve of the residuals over time, and the dashed lines indicate ±2 standard errors. No systematic deviation from zero was observed, indicating that the PH assumption was not violated for either group (Global PH test p = 0.587).

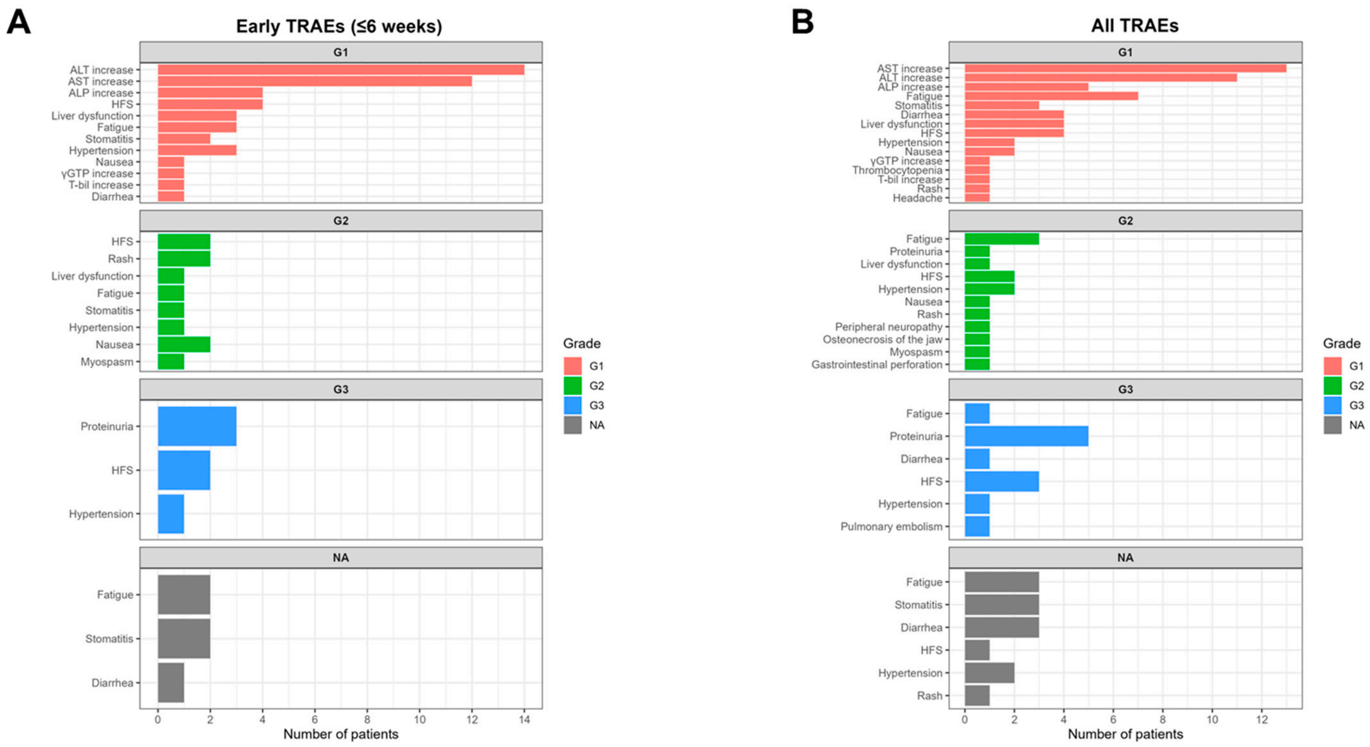

Figure S4. Treatment-related adverse events **(A)** Early treatment-related adverse events (TRAEs, ≤6 weeks) in patients treated with cabozantinib. Each bar represents the number of patients who experienced a specific TRAE within the first 6 weeks of treatment. Events are categorized by grade according to Common Terminology Criteria for Adverse Events v5.0 (G1–G3, NA = grade not specified). The most frequent early TRAEs were liver enzyme elevations (ALT/AST increase, G1), palmar–plantar erythrodysesthesia (HFS, G1), and proteinuria (G3). **(B)** All treatment-related adverse events (TRAEs) during cabozantinib therapy. Bar plots show the cumulative number of patients who experienced each TRAE at any time during cabozantinib therapy, stratified by grade. The most common TRAEs overall included liver enzyme elevations (AST/ALT, G1), fatigue (G1), and proteinuria (G3).

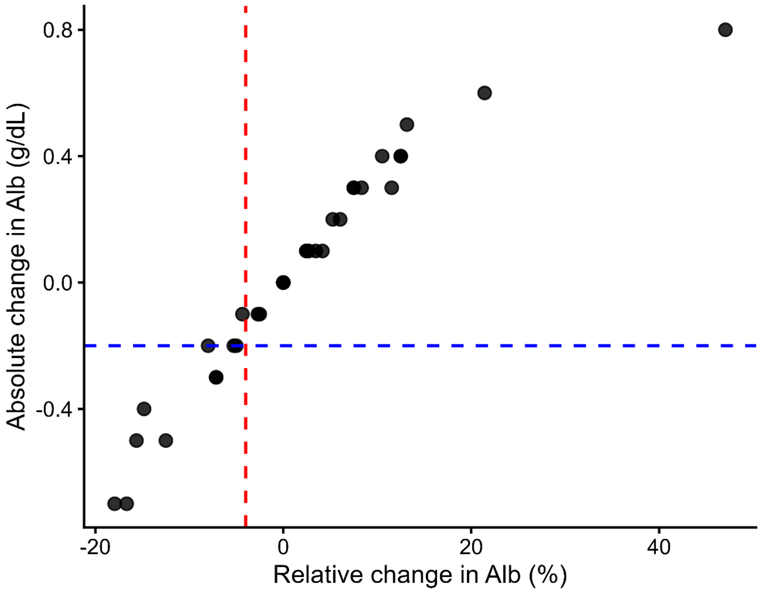

Figure S5. Relationship between relative and absolute changes in serum albumin (Alb). Scatter plot showing correlation between absolute change in Alb (g/dL) and relative change in Alb (%). Venn diagram summarizing patients fulfilling relative (≤ −4%) and absolute (≤ −0.2 g/dL) criteria.

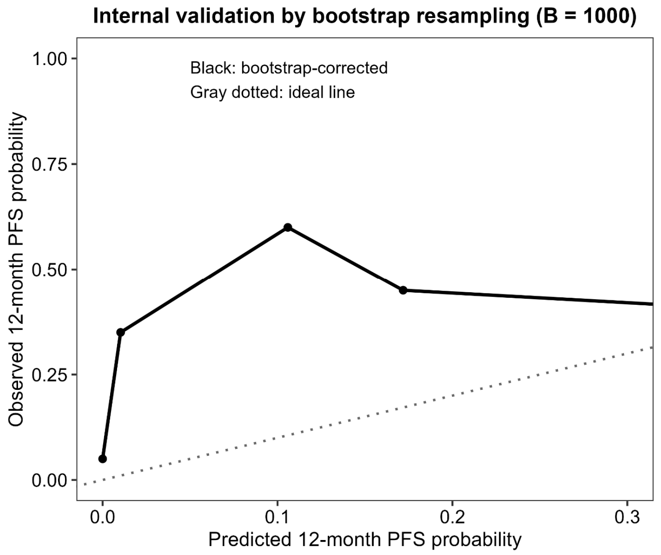

Figure S6. Internal validation of the multivariable Cox proportional hazards model by bootstrap resampling (B = 1000). Calibration of the predicted 12-month progression-free survival (PFS) probability. The black solid line indicates the apparent performance of the original model, while the blue dashed line shows the bootstrap-corrected estimates. The gray dashed diagonal line represents

perfect calibration (predicted = observed). The calibration curve demonstrates acceptable agreement between predicted and observed probabilities, indicating adequate internal validity of the model.
